# A Cancer Patient Journey: Complete Review During Acute Treatment Phase

**DOI:** 10.1089/heq.2019.0046

**Published:** 2019-08-12

**Authors:** Saima Siddiqui, Inez Cruz

**Affiliations:** Department of Family and Community Medicine, University of Texas Health Science Center at San Antonio, San Antonio, Texas.

**Keywords:** qualitative research, cancer, health, shared care, primary care

## Abstract

**Purpose:** Cancer is a chronic illness with acute episodes lasting for years. Most cancer patients have coexisting comorbidities, which affect cancer treatment outcomes and make a shared care model for chronic diseases essential. There is a considerable gap between the achievable and delivered quality of care for cancer patients.

**Methods:** We used a case study approach to examine the complexity of cancer management, from the perspective of one person's case as interpreted by the care team. It allowed the complexity of cancer management to retain its holistic and meaningful characteristics. We interviewed the patient, caregiver, primary care physician (PCP), and oncologist. Interviews were audio recorded and analyzed with ATLASti, qualitative statistical software. Participants also completed a basic demographic survey. Common themes were identified, analyzed, and discussed.

**Results:** Main themes were lack of longitudinal relationship with PCP, communication barriers, and ambiguous health care provider roles. Communication barriers can be associated with the other two main themes.

**Conclusion:** Our results showed that shared care for cancer management is lacking during the acute cancer treatment phase. Communication barriers between the PCP and oncologist along with lack of continuity of care and unclear role of the PCP are major contributors for fragmented cancer care in U.S. health care system.

## Introduction

Cancer is a leading cause of morbidity and mortality worldwide. In 2013, there were ∼1,660,290 new cancer cases and 580,350 cancer deaths in the United States.^1^ A diagnosis of cancer is still very stressful and frightening for the majority of patients and families^2–4^ although the number of patients living with cancer has increased threefold since 1971.^5^ According to the Center for Disease Control and Prevention (CDC), cancer is classified as a chronic disease and described as of long duration and generally slow progression.

Studies have shown that cancer patients receive fragmented care especially during the acute treatment phase^6–9^ stemming from system problems such as lack of health insurance, communication problems between health care team members,^7,10^ and lack of role clarity among team members^11–14^ The National Cancer Policy Board has concluded that for many Americans with cancer, there is a wide gulf between what could be construed as the “ideal and the reality of their experience with cancer care.”^15^ There is not just a “gap, but a chasm.”^16^

The purpose of this study was to gather a qualitative description of collaborative care from viewpoints of cancer patient (Pt), caregiver, primary care physician (PCP), and oncologist to better understand cancer patient's journey in the health care system.

## Methods

A case study approach was used because it allowed us to examine the complexity of cancer management from the perspective of one person's case as interpreted by multiple people, retaining its holistic and meaningful characteristics while being studied^17^ answering how and why questions.^18^ Interviews from four participants presented multiple perspectives of the same interested topic, therefore achieving data triangulation.

The study is guided by the chronic care model (CCM), a proactive approach to keep patient healthy through productive collaboration between community and health systems; therefore, the study generalizes to the theoretical propositions of the CCM and not the population.^19^ CCM identifies six structural elements: the community, the health system, self-management support, delivery system design, decision support, and clinical information systems. The interview guide was developed based on these areas. Development of the interview guide was an iterative process in which the researchers developed and discussed questions, which contextualized the CCM within cancer management. Once questions were approved, they became part of the official interview guide (12 total questions), which was re-evaluated for consistency and changed if researchers felt that questions were being misinterpreted. Main questions included were as follows: Tell me the story of how you learned you had cancer? Who did you talk to about your cancer diagnosis? How do your PCP and oncologist work together to manage your cancer and general medical care? The researcher conducting the interviews was knowledgeable in social science research of chronic conditions. Her inexperience served as a strength because she was not able to create leading questions or force participants into expected outcomes.

The study utilized a purposeful sampling method.^20,21^ PCP identified and referred the cancer patient to the study. The patient then identified her caregiver and oncologist.

Data collection consisted of about 1 h long semistructured interview. The approach of starting with the patient and then interviewing the caregiver and the clinicians helped to understand how the same events were viewed from different perspectives. Each interview was recorded and transcribed for analysis. All research activities were reviewed and approved by the University of Texas Health Science Center San Antonio Office of Institutional Review Board.

## Results

Our sample included interviews with one cancer patient, her caregiver, PCP, and oncologist. Specifics of sample demographics are in Tables 1 and 2).

**Table 1.** Demographics of Patient and Caregiver in Case Study

Role	Sex	Age	Ethnicity	Cancer	Insurance	Marital status	Education	Monthly income
Patient	F	63	Hispanic	Leukemia	County system	Divorced	GED	<$1000
Caregiver	F	31	Hispanic	n/a	Private	Married	College graduate	$2500–3500

F, female.

**Table 2.** Demographics of Providers in Case Study

Role	Sex	Ethnicity	Specialty	Years of experience
PCP	F	White	Family medicine	25
Oncologist	M	Asian	Hematology	29

M, male; PCP, primary care physician.

The results identified a major breakdown in the delivery system design highlighted in the CCM. The three main themes, which are organized around the patients' experience through the health care system—from cancer diagnosis to treatment, are as follows:

(a) Lack of longitudinal relationship with PCP
(b) Communication barriers
(c) Ambiguous health care provider role.

*(a) Lack of longitudinal relationship with PCP:* This theme supports a known system problem in which people who are the sickest and need health insurance the most do not have it. This particular patient lost insurance, secondary to unemployment because of uncontrolled hypertension.

The patient ended up in the emergency room (ER) for severe chest pain and was diagnosed as having leukemia.

**Pt:** “At that time, I did not [have a PCP] … I was one of those persons that go from payday to payday and I could not afford health insurance…Here I am very sick, quitting because I am very sick…So when I went into ER, I did not have a doctor…”

The first PCP visit was 4 months after her leukemia diagnosis and after receiving three cycles of chemotherapy.

**Pt:** “After my fourth visit to the [cancer treatment center] they told me that I need to call the [healthcare system] and I needed to get a PCP…”

The patient was assigned a new PCP in a teaching facility with residents and faculty members divided into teams and different providers saw the patient every time. The lack of a longitudinal relationship with a PCP appeared normal to the patient; therefore, she began to rely heavily on the oncology team and ER for things that a PCP could manage.

**Pt:** “Usually I see a different [provider], it is like a set of doctors that all work together. So, I can't say it is one doctor…”

*(b) Communication barriers:* The communication barriers surrounding cancer treatment began between the patient, caregiver, and health care providers almost immediately. As the patient was diagnosed with cancer in the ER, she felt she was not able to get the answers for her questions,

**Pt:** “The whole time all I was thinking, I have cancer! What is Leukemia… am I going to die… I heard them talking between themselves that it might be Leukemia…[And] they didn't want to give me the exact diagnosis yet.”

Similarly, the caregiver was not included in any stage of cancer management. After a few months, the oncologist provided a video to share with family members.

**Caregiver:** “..a lot of questions I had, I just used my own resources… [the providers asked] if you have questions…and then they gave us some packets and pamphlets. I relied mostly on my mom for communication… My mom has a high school education, GED, and she doesn't understand lots of words.”

The teaching practice setting also prevented her from communicating with the health care team. The caregiver's impression was that due to patient's privacy, physicians were not supposed to communicate with her, and residents' learning will be interrupted by her questions.

**Caregiver:** “I always assume, it's a privacy thing… I just wish there was a means for me to communicate directly with them or staff or nurse. I feel like there's residents that come in as a group with the doctors and learn, so I feel like I didn't want to interrupt their learning with questions.”

Caregiver also identified lack of communication between the PCP and oncologist.

**Caregiver:** “I feel like there's a lack of communication between them. That's a prescription given to her by her cancer doctor. Then the PCP will say that's not working out for you, let's take them off so that makes me uncomfortable, just in the sense that I feel like you should ask [the oncologist] first… My mom's been bounced back and forth between vitamins and medications that she'll get prescribed by one doctor, and then another doctor will change their mind..”

There was no specific arrangement for cancer patients to contact PCP for early or urgent appointments. When the caregiver called to report a concern, appointment clerk asked her to go to the ER. At times, the ability to provide advice was contingent on the flow of clinic traffic, sometimes the patient and caregiver were able to contact the oncologist but got same advice.

Both the PCP and oncologist identified lack of communication as a barrier for patient management. The health care providers were not able to effectively communicate because of the distance between facilities, a physical difficulty, and relational issue.

**Oncologist**: “The physical issue of being based in a downtown [building] and having oncology services out at medical center [approximately 12 miles away]. You can't pop over at lunch for a meeting ever, I suppose..”

Time constraint was another factor. PCPs do not have time to serve on cancer boards and oncologists do not communicate with PCP by phone or with follow-up letters.

**Oncologist:** “No one's going to serve on a board if they're all in clinic full time, of course… Everyone's busy so the communication is lacking…because we in oncology have been very short staffed.”

Use of different electronic health record (EHR) systems break communication further.

**PCP:** “It is not possible for providers to look into each other notes and management plan. You've got the problem of the two computer systems that don't talk to each other, so they don't see what they are doing in [EHR] and we don't see what they are doing in their EHR, so that makes it very difficult just all around.”

No point of contact within the PCP and oncology clinic was assigned for communication about patients. The PCP was expected to communicate through the oncology on-call resident or the front desk person for any question or concern. It resulted in duplicate laboratory tests and confusion about patient's treatment and patient served as the main communicator between the PCP and oncologist. It also resulted in care delay.

**PCP:** “These are all unnecessary barriers in communication between the two offices and one of them is the fact that you can't just book the patient before the patient leaves… sometimes you think you are conveying information, sometimes they don't receive it.”

*(c) Ambiguous health care provider role:* The patient, caregiver, and health care providers agree that the PCP should be an essential part of the management team; however, all ambiguously understands the role. The PCP was viewed as important for the emotional support of the patient and family.

**Pt:** “She [the PCP] asked me if I ever got depressed. I told her no… She said, it is okay to say it if you are…I told her when I get in the shower I just breakdown crying sometimes for no reason. She says good, let it out. She says it is okay to feel that way. I would feel that way too.”

However, the PCP was not comfortable in managing specific chemotherapy-related side effects. The PCP felt that their strengths were in management of chronic diseases. The oncologist felt that they should be able to rely on the PCP for support of common disease management.

**Oncologist:** “..honestly my knowledge of ideal hypertension management has declined… even though I am an internist at heart… I quickly need primary care support to manage hypertension, as well as routine health maintenance of immunizations and recommended cancer screenings. We [the oncologist] make the diabetes worse, so we constantly want to work with primary care teams.”

The oncologist identified that the PCP should be seen as **(Oncologist):** “an educator or tie-breaker in terms of treatment decision making.”

## Discussion

This study reflects a typical journey of an underprivileged uninsured cancer patient through the American health care system. It is unique in that data were collected and interpreted from the patients' perspective but captures several perspectives on the experience. No other studies, focusing on the patient perspective from a case study methodology, were found in the current literature. The patient lost her health insurance due to uncontrolled hypertension resulting in the loss of employment. This resulted in a delay of cancer diagnosis as the patient kept on postponing and neglecting the symptoms as long as she could tolerate. Main barriers identified in our study were the same as identified in earlier studies. Similarities included a lack of longitudinal relationships with the PCP, communication issues between patient, caregiver, PCP, and oncologist, and a lack of role clarification for providers and patient.^14,22–25^ New finding was the patient and caregiver's inability to communicate with PCP due to the teaching practice setting.

This study identifies the serious gaps and areas of improvement for cancer. Our findings confirm that the PCP is not an active member of patients' management team during chemotherapy.^25–27^ The first PCP visit took place after the fourth chemotherapy visit, ∼3½ months into chemotherapy. Studies have shown that one in five Americans reported not getting or delaying medical care, and the percentage of uninsured patients 45–64 years of age increased from 13.1% to 15.6%.^28,29^ In addition, the patient did not have access to a PCP after obtaining health care insurance due to the PCP's busy schedule and the absence of special arrangements for cancer patients, which resulted in patients using the ER. Previous studies have shown that there is an increased use of health care services by cancer patients when they are undergoing acute cancer treatment by chemotherapy and radiation as well as after treatment.^30,31^ Ideally, there needs to be special provisions or the identification of a key contact person for cancer patients in PCP offices.

Lack of communication was the most prominent problem identified by the patient, caregiver, and physicians. The main communication failure was between the PCP and the oncology team, confirming similar findings identified in other studies.^32–36^ The federal government has offered incentives for meaningful use of information technology as a key tool for improving care coordination, which resulted in an increased use of EHR by physicians and hospitals.^37,38^ In our study, the use of different EHRs by the oncologist and the PCP office was problematic. The PCP could not access patient information from the oncology visit and there was no formal follow-up letter from oncology. Therefore, the PCP did not have any idea about chemotherapy regime or patient prognosis. Ideally, EHR should account for human factors both tolerating human limitations and augmenting human strengths,^39^ and bridging the gap between different segments of patient care rather than collecting numbers and producing reports to fulfill government requirements.

Similarly, the patient and caregiver expressed frustration about the lack of communication because it placed a larger burden on the patient as main communicator between oncologist and PCP, which is not an acceptable practice.

Not knowing the point of contact in the PCP and oncology office was an additional reason for communication breakdown. Good care coordination for safe and appropriate management of chronic conditions such as cancer are essential, but the care coordination remains inadequate and a major cause of health care expenditure and mistakes.^40,41^ Possible solutions include uniform access to EHRs, clear identification of the patient's PCP and oncologist, a point of contact in each office, and a structured follow-up letter from oncologist to PCP.^42^ Further studies are needed to evaluate the efficacy of these measures.

Time constraint was an additional reason identified by the PCP and oncologist for the communication breakdown. There is no formal reimbursement for physician or staff time used for communication and coordination between providers or by insurance companies.^43^ In addition, the shortage of PCPs and oncologists, and increased number of cancer patients makes care coordination more difficult.^44^ A system-wide change is needed to address these issues and acknowledge that time reimbursement will produce real improvement in patient care and reduce health care cost. The patient and caregiver identified the teaching hospital setting as an inhibitory factor because they felt that asking questions and communicating with health care providers would interfere with learning, which is a new finding by this study. It requires that teaching physicians take extra steps to include the patient and care giver in their discussions and make them feel like part of the team by formally including the patient in discussions.

Lack of PCP role clarification was another barrier identified for effective collaboration.^45–47^ The current norm accepts that PCPs will not be a part of the cancer patient health care team. The patient and caregiver expectations were that the PCP would serve as emotional support, manage chronic disease, and perform routine health maintenance such as cancer screening and immunizations. Studies have shown that PCPs can play an important role in the management of cancer patients' coexisting chronic conditions and common side effects of chemotherapy, treating acute conditions such as viral illnesses and helping patient to make informed decisions about management, and end-of-life issues.^48^ The oncologist agreed that the PCP was an important part of the health care team, and the PCP was comfortable in fulfilling all these roles. Clear role assignment of health care team members will decrease the role confusion and potentially impact patients' unnecessary ER visits, reducing patient discomfort and health care cost.

Our study revealed many barriers for collaboration during the initial cancer treatment phase between the PCP and oncologist. Even though there is an abundance of resources and expertise available, the lack of collaboration and fragmented effort resulted in a wide gap between possible and actual care delivery for cancer patients.

### Limitations

The major weaknesses of this study are that it was conducted in a teaching hospital setting and describes the experience of only one patient. However, the purpose of a case study was to examine the complexity of a phenomenon (cancer management) while it retains its holistic and meaningful characteristics. Major strength of this study is that it describes the complete experience, as it has been understood by an underserved and uninsured patient, caregiver, and patient health care team.

### Implications

The barriers identified in this study should be used to devise interventions to be tested in large-scale prospective studies to fill gaps in present system of cancer patient care.

## Abbreviations Used

CCM: chronic care model
CDC: Center for Disease Control and Prevention
ER: emergency room
EHR: electronic health record
PCP: primary care physician
